# Supplementary material for: Predicting visual function by interpreting a neuronal wiring diagram
Source: Nature. 2024 Oct 2;634(8032):113–23. doi: 10.1038/s41586-024-07953-5 (PMC11446822; doi:10.1038/s41586-024-07953-5)
Supplement: Supplementary file 6 — CRF and ERF predictions for individual TmY4 and TmY9 cells. Analogous to Supplementary Data 3, but for TmY target types. Shown are the top four monosynaptic pathways, the strongest pathway passing through each of the top ten intermediary types (ranking from Extended Data Fig. 7), and the trisynaptic pathway Tm1–TmY–Dm3–TmY (see the section entitled Prediction of spatial normalization). [file 41586_2024_7953_MOESM6_ESM.zip › DataS4/TmY9qΓèÑ/720575940627636562.pdf]

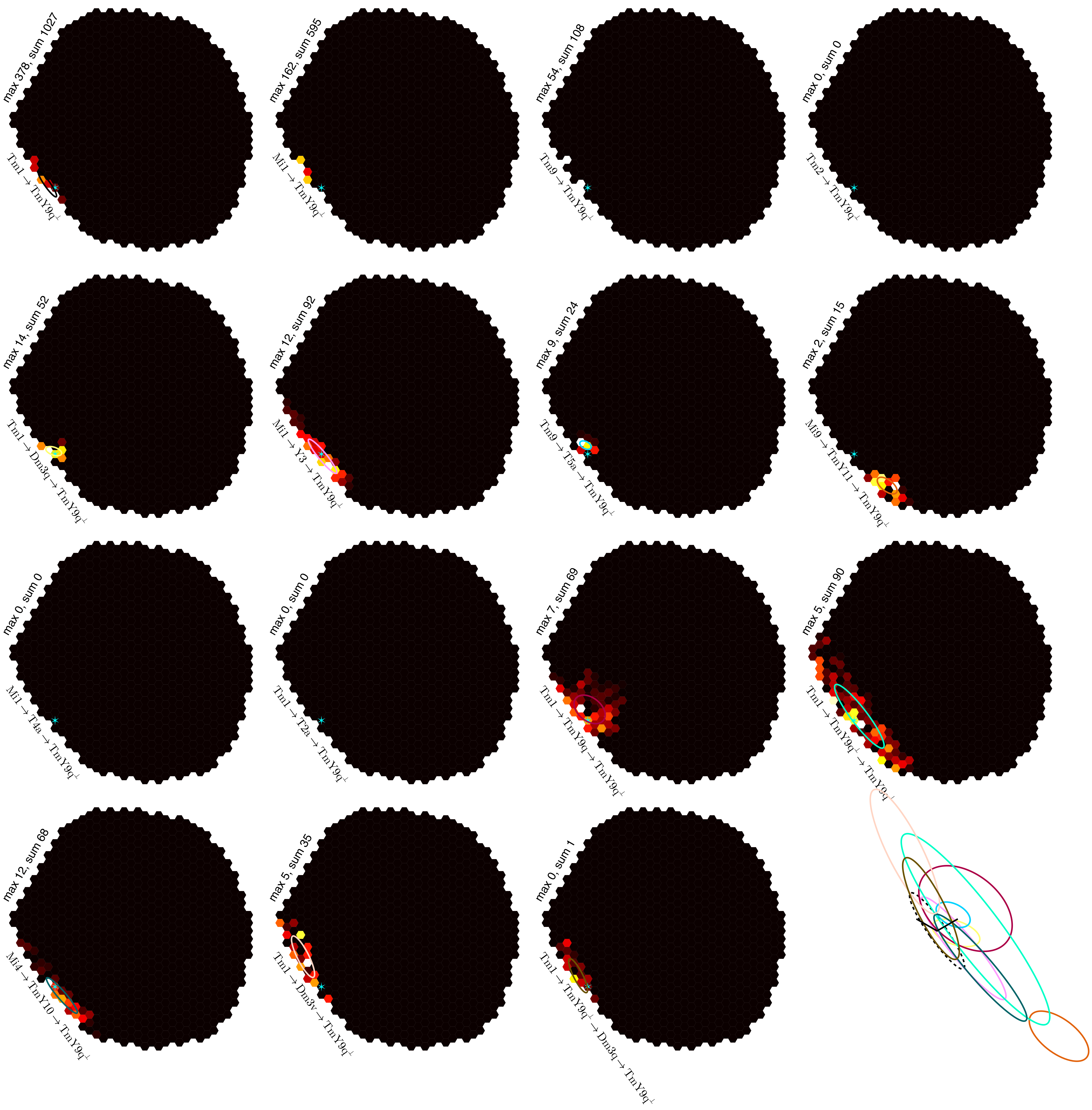

max 378, sum 1027
Tm1 → TmY9q⊥
max 162, sum 595
Mi1 → TmY9q⊥
max 54, sum 108
Tm9 → TmY9q⊥
max 0, sum 0
Tm2 → TmY9q⊥
max 14, sum 52
Tm1 → Dm3q → TmY9q⊥
max 12, sum 92
Mi1 → Y3 → TmY9q⊥
max 9, sum 24
Tm9 → T5a → TmY9q⊥
max 2, sum 15
Mi9 → TmY11 → TmY9q⊥
max 0, sum 0
Mi1 → T4a → TmY9q⊥
max 0, sum 0
Tm1 → T2a → TmY9q⊥
max 7, sum 69
Tm1 → TmY9q⊥ → TmY9q⊥
max 5, sum 90
Tm1 → TmY9q⊥ → TmY9q⊥
max 12, sum 68
Mi4 → TmY10 → TmY9q⊥
max 5, sum 35
Tm1 → Dm3v → TmY9q⊥
max 0, sum 1
Tm1 → TmY9q⊥ → Dm3q → TmY9q⊥
